# Validation of biomarkers to predict response to immunotherapy in cancer: Volume I — pre-analytical and analytical validation

**DOI:** 10.1186/s40425-016-0178-1

**Published:** 2016-11-15

**Authors:** Giuseppe V. Masucci, Alessandra Cesano, Rachael Hawtin, Sylvia Janetzki, Jenny Zhang, Ilan Kirsch, Kevin K. Dobbin, John Alvarez, Paul B. Robbins, Senthamil R. Selvan, Howard Z. Streicher, Lisa H. Butterfield, Magdalena Thurin

**Affiliations:** 1 Department of Oncology-Pathology, Karolinska Institutet, 171 76 Stockholm, Sweden; 2 NanoString, Inc, 500 Fairview Avenue North, Seattle, WA 98109 USA; 3 Nodality, Inc, 170 Harbor Way, South San Francisco, 94080 CA USA; 4 ZellNet Consulting, Inc, 555 North Avenue, Fort Lee, 07024 NJ USA; 5 Covaris Inc, 14 Gill St, Woburn, MA 01801 USA; 6 Adaptive Biotechnologies, Inc, 1551 Eastlake Ave. E, Seattle, WA 98102 USA; 7 Department of Epidemiology and Biostatistics, College of Public Health, The University of Georgia, 101 Buck Road, Athens, 30602 GA USA; 8 Janssen Research & Development, LLC, Spring House, PA 19477 USA; 9 Pfizer, San Diego, CA USA; 10 Omni Array Biotechnology, 15601 Crabbs Branch Way, Rockville, 20855 MD USA; 11 National Cancer Institute, National Institutes of Health, 9609 Medical Center Drive, Bethesda, 20892 MD USA; 12 Department of Medicine, Surgery and Immunology, University of Pittsburgh Cancer Institute, 5117 Centre Avenue, Pittsburgh, PA 15213 USA; 13 National Cancer Institute, Cancer Diagnosis Program, DCTD, National Institutes of Health, 9609 Medical Center Drive, Bethesda, 20892 MD USA

**Keywords:** Biomarker, Immunotherapy, Cancer, Assay, Validation, Standardization

## Abstract

Immunotherapies have emerged as one of the most promising approaches to treat patients with cancer. Recently, there have been many clinical successes using checkpoint receptor blockade, including T cell inhibitory receptors such as cytotoxic T-lymphocyte-associated antigen 4 (CTLA-4) and programmed cell death-1 (PD-1). Despite demonstrated successes in a variety of malignancies, responses only typically occur in a minority of patients in any given histology. Additionally, treatment is associated with inflammatory toxicity and high cost. Therefore, determining which patients would derive clinical benefit from immunotherapy is a compelling clinical question.

Although numerous candidate biomarkers have been described, there are currently three FDA-approved assays based on PD-1 ligand expression (PD-L1) that have been clinically validated to identify patients who are more likely to benefit from a single-agent anti-PD-1/PD-L1 therapy. Because of the complexity of the immune response and tumor biology, it is unlikely that a single biomarker will be sufficient to predict clinical outcomes in response to immune-targeted therapy. Rather, the integration of multiple tumor and immune response parameters, such as protein expression, genomics, and transcriptomics, may be necessary for accurate prediction of clinical benefit. Before a candidate biomarker and/or new technology can be used in a clinical setting, several steps are necessary to demonstrate its clinical validity. Although regulatory guidelines provide general roadmaps for the validation process, their applicability to biomarkers in the cancer immunotherapy field is somewhat limited. Thus, Working Group 1 (WG1) of the Society for Immunotherapy of Cancer (SITC) Immune Biomarkers Task Force convened to address this need. In this two volume series, we discuss pre-analytical and analytical (Volume I) as well as clinical and regulatory (Volume II) aspects of the validation process as applied to predictive biomarkers for cancer immunotherapy. To illustrate the requirements for validation, we discuss examples of biomarker assays that have shown preliminary evidence of an association with clinical benefit from immunotherapeutic interventions. The scope includes only those assays and technologies that have established a certain level of validation for clinical use (fit-for-purpose). Recommendations to meet challenges and strategies to guide the choice of analytical and clinical validation design for specific assays are also provided.

**Electronic supplementary material:**

The online version of this article (doi:10.1186/s40425-016-0178-1) contains supplementary material, which is available to authorized users.

## Background

Increased understanding of cellular and molecular tumor immunology over the past two decades has enabled the identification of new ways to manipulate the immune response against cancer to counteract immunosuppressive mechanisms that evolve during tumor progression. Monoclonal antibodies (mAbs) to the cytotoxic T-lymphocyte-associated antigen 4 (CTLA-4) and programmed cell death-1 (PD-1) protein, two T cell-inhibitory checkpoint receptors with independent mechanisms of action, have demonstrated improvement in overall survival in advanced melanoma patients [1–3]. Significant clinical benefit (including durable tumor responses and extension of progression-free and overall survival) has also been shown in tumor types as diverse as non-small cell lung cancer (NSCLC), renal cell carcinoma (RCC), bladder cancer, and Hodgkin’s disease [4–12].

Despite demonstrated successes, responses to immunotherapy interventions only occur in a minority of patients. Attempts are being made to improve the activity of immunotherapies with novel combinatorial strategies and with biomarker optimization. A wave of recent clinical trial results has highlighted the potential for combination therapies that include these immunomodulating agents [13–17]. A wide range of biomarkers and assays is required to guide cancer therapy for several reasons: i) a variety of immunotherapy agents with different mechanisms of action including immunotherapies that target activating and inhibitory T cell receptors (e.g., CTLA-4 and PD-1), adoptive T cell therapies that include tissue infiltrating lymphocytes (TILs), chimeric antigen receptors (CARs), and T cell receptor (TCR) modified T cells [18]; ii) tumor heterogeneity including changes in antigenic profiles over time and location for an individual patient; and iii) a variety of immune-suppressive mechanisms that are active in the tumor microenvironment (TME). Optimizing biomarkers for immunotherapy could help to properly select patients for treatment, identify rational combination therapies, and define progression and resistance. In addition, biomarkers may help define the mechanism of action for different agents and help with dose selection as well as sequencing of drug combinations. Although most of immune therapies engage T cells and the assessment of cell-mediated cytotoxicity is integral for the selection of biomarkers of response to immunotherapy, the cancer immune response is a multi-step process involving interactions between the tumor and microenvironment including multiple cell subsets and soluble mediators functioning at different times and at different anatomical sites (tumor, lymph nodes, and blood) as well as the tumor stroma and vasculature. Thus, profiling of the tumor-immune interface with multiparametric technologies that encompass the dimensionality and complexity of this interaction are likely to be needed to monitor and stratify cancer patients for individual therapeutic requirements.

A number of candidate biomarkers and platforms with the potential to be developed into assays to predict response to immunotherapy have been identified in research studies. Platforms based on multiplexed transcriptome analysis, protein expression, and genomic variability are discussed in SITC Immune Biomarker Task Force reports (Additional file 1). The availability of these platforms and novel technologies should facilitate the integration of the molecular features of the tumor and the host factors for the development of multiplex profiles to guide personalized treatment in the future.

The focus of this review is to discuss the requirements for advancing a biomarker assay through the validation process to its clinical application. The validation of such assays should ultimately qualify them for use in clinical decision making. Specific examples of the assays already in use such as immunohistochemistry (IHC) based PD-L1 assays or soon be approved for use in clinical laboratories are discussed to illustrate the requirements for analytical validation (Table 1). Prototypes of these assays have been shown in research and small clinical studies  to be potentially useful as patient enrichment tools. Although analytical validation data for each specific platform are available, none of these have been clinically validated yet as a predictive biomarker, except for PD-L1, which will be discussed below.

**Table 1:** Examples of cancer biomarker assays predictive of response to immunotherapy with different levels of evidence for clinical validity/utility

Biomarker Assay	Biomarker	Clinical Use	Study Type/Level of Evidence	References/ Regulatory Clearance
IHC, PD-L1 22C3 pharmDx, Companion Diagnostic	PD-L1	Predicting response to anti-PD-1 therapy (pembrolizumab) in NSCLC 50 % cut-off	Prospective, Phase III clinical trial KEYNOTE-001	FDA approval [32]
IHC, PD-L1 28-8 pharmDx, Complementary Test	PD-L1	Informs about risk vs. benefit of anti-PD-1 therapy (nivolumab) in non-squamous NSCLC and melanoma- continuous correlation of PD-1 expression with magnitude of treatment effect	Prospective-retrospective, Phase III clinical trial CheckMate-057	FDA approval [33]
IHC, PD-L1 SP142, Complementary Test	PD-L1	Informs about the risk vs. benefit of anti-PD-L1 therapy (atezolizumab) for metastatic urothelial bladder cancer	Prospective-retrospective, Phase II clinical trial IMvigor-210	FDA approval [34]
IHC	Tumor T cell Infiltrate, PD-L1 with spatial resolution	Predictive to anti-PD-1 therapy in melanoma and NSCLC	Retrospective, Exploratory analysis	Tumeh et al., 2014 [6]; Teng et al. 2015 [35]; Herbst et al., 2014 [36]
Enzyme Linked Immunospot (ELISpot)	IFNγ release	Post-treatment/monitoring, cancer vaccines	Retrospective, Exploratory analysis	Kenter et al., 2009 [21]; Sheikh et al., 2009 [24]
Multi-parametric Flow Cytometry	MDSC, Tregs, ICOS+ CD4 T cells	Post-treatment/monitoring, cancer vaccines, Predictive of anti-CTLA-4 therapy in RCC and melanoma	Retrospective, Exploratory analysis, Phase I,II trial	Walter et al., 2012 [22]; Tarhini et al., 2014 [171]; Di Giacomo et al., 2013 [172]; Hodi et al., 2014 [173]; Martens et al., 2016 [20]
Multi-parametric Flow Cytometry	Absolute lymphocyte count (ALC)	Predictive of response to anti-CTLA-4 therapy	Retrospective, Small cohort, Significant variability among institutions	Ku et al., 2010 [174]
Single Cell Network Profiling (SCNP)	AraC → cPARP AraC → CD34	Predictive of response to induction therapy in elderly patients with *de novo* acute myeloid leukemia	Retrospective, Training and validation study establishing clinical utility	Cesano et al., 2015 [29]
TCR Sequencing	Limited clonality	Clonality assessments of tumor-infiltrating lymphocytes, Predictive to response with anti-CTLA-4 and anti-PD-1 in melanoma	Retrospective, Small cohort	Tumeh et al., 2014 [6]; Cha et al., 2014 [59]
nCounter Gene Expression, NanoString Technologies, Inc.	Gene expression profile	Predictive of response to anti-PD-1 therapy in melanoma and multiple solid tumors	Retrospective, Training and test sets – prospective validation ongoing on different tumor types	Ribas et al., 2015 [61]; Wallden et al., 2016 [175]; Piha-Paul et al., 2016 [176]; Man Chow et al., 2016 [177]
Next Generation Sequencing (NGS)	Mutational load	Predictive of response to anti-CTLA-4 therapy in melanoma and anti-PD-1 in NSCLC	Retrospective, Small cohort, Training and test sets	Snyder et al., 2014 [46]; Rizvi et al., 2015 [47]
NGS/in silico Epitope Prediction	MHC class I epitope frequency/specificity	Predictive of response to anti-CTLA-4 and anti-PD-1 in melanoma, NSCLC, and CRC	Retrospective, Small cohorts	Snyder et al., 2014 [46]; Rizvi et al., 2015 [47]; Van Allen et al., 2015 [52]; Van Rooij et al., 2013 [48]
IHC or PCR, Microsatellite Instability Analysis	Mismatch-repair status	Predictive of response to anti-PD-1 therapy in CRC	Phase II study, small cohort	Le et al., 2015 [53]

According to the position paper by Lee and colleagues [19], the biomarker assay validation process can be separated into several continuous steps: assessment of basic assay performance (analytical validation); characterization of the performance of the assay with regard to its intended use (clinical validation); and validation in clinical trials that ensures that the assay performs robustly according to predefined specifications (fit-for-purpose) and facilitates the establishment of definitive acceptance criteria for clinical use (validation of clinical utility). The fit-for-purpose approach for biomarker development and validation addresses the assay validation that should be tailored to meet the intended purpose of the biomarker. The fit-for-purpose method validation is an umbrella term that is used to describe distinct stages of the validation process.

Specifically,

- Analytical validation defines how accurately and reliably the test measures the analyte(s) of interest in the patient specimen. Analytical validity is defined as the assay’s ability to accurately and reliably measure the analyte of interest in the clinical laboratory and in specimens representative of the population of interest. Analytical validity refers to the three different phases of assay development: pre-analytical, analytical, and post-analytical phase.
- Clinical validation should demonstrate how robustly and reliably the test results correlate with the clinical outcome of interest. Practically, clinical validity implies that the cancer biomarker assay separates a population into two or more distinct groups with different biological characteristics or clinical outcomes.
- Clinical utility is defined as an assay’s ability to significantly improve clinical outcomes, i.e., does the use of the biomarker result in patient benefit or add value to patient management decision making compared with current practices.

Pre-analytical and analytical assay validation steps are discussed in Volume I, while Volume II is focused on the clinical validation and validation of clinical utility of the assays as well as regulatory considerations.

### Assays examples

Specific examples of relevant assays are discussed in detail in the following section and are summarized in Table 1. The scope of the paper includes only those assays that have established a certain level of validation for clinical use as biomarkers predictive of response to immunotherapy. Multiple biomarkers and platforms that require standardized assays and are lacking even initial clinical validation demonstrating its clinical utility (fit-for-purpose) are not the focus of this publication.

#### 1. Flow cytometry

Phenotypic analysis of T cells can provide information regarding their activation status using assays based on multiplex flow cytometry examining a panel of lymphocyte markers. A baseline signature of frequencies of myeloid-derived suppressor cells (MDSCs) and regulatory T cells (Tregs), and high absolute eosinophil counts (AEC) has been recently shown to be associated with favorable outcome in patients with melanoma receiving ipilimumab [20]. Interestingly, higher baseline frequencies of circulating CD4 + CD25 + FoxP3+ Tregs were associated with improved overall survival (OS) in this patient population [20]. Tregs represent direct target cells of ipilimumab due to constitutive expression of CTLA-4 by those cells which might be one of the reasons that patients with higher levels of circulating Tregs are more likely to benefit from anti-CTLA-4 antibodies. In order to be implemented in routine clinical settings, this biomarker signature needs to be analytically and clinically validated (including a panel of markers required for the analysis and enumeration of MDSCs and Tregs) [20].

#### 2. Enzyme-linked ImmunoSpot (ELISpot)

Enzyme-Linked ImmunoSpot (ELISpot) is a highly quantitative assay for monitoring the secretion of cytokines and cytotoxic mediators (e.g., perforin, granzyme B). It can measure a wide range of cellular responses and is capable of assessing critical immune-related activity of antigen-specific T cell stimulation. The most common analytes investigated today are cytokines (interferon (IFN)ɣ, interleukin (IL)-2, IL-5, IL-10, IL-17, granzyme B, tumor necrosis factor (TNF), and granulocyte-macrophage colony-stimulating factor (GM-CSF)). Other factors can also be evaluated with this platform, such as chemokines (e.g., CXCL8, CCL4). The IFNγ ELISpot assay has been used extensively for monitoring immune responses in the development of vaccines for the prevention and treatment of infectious diseases; however, there is also a body of literature demonstrating the correlation of the clinical outcome of cancer patients in immunotherapeutic trials with ELISpot results [21, 22].

Specifically, clinical trials have shown a significant correlation of antigen-specific ELISpot responses with patient survival after administration of a melanoma antigen-specific peptide based vaccine in advanced-stage patients [23]. The magnitude of antigen-specific IFNγ-secreting cells, as measured by ELISpot, showed correlation towards survival after the administration of a prostate-specific antigen vaccine in prostate cancer patients, as well as a human epidermal growth factor receptor 2 (HER2/neu) specific vaccine in breast cancer patients [24–26]. Compared to the IFNγ ELISpot assay, the granzyme B ELISpot may be a more direct measure of cytotoxic cell activity because it measures one of the primary effector molecules of cell-mediated cytotoxicity. Cytotoxic activity of CD8+ T cells measured by granzyme B release after stimulation with MUC antigen was found to be predictive for the survival of pancreatic cancer patients independently of type of therapy (chemoradioimmunotherapy or 5FU-based chemotherapy) [27]. Considering that the tumor infiltration is a reflection of a pre-existing immunity and is predictive of response to anti-checkpoint immunotherapy (discussed below), it appears logical to assume that functional assessment of cytotoxic activity of CD8+ T cells following stimulation with specific tumor associated antigen(s) by ELISpot may also be predictive of response to immunotherapy.

#### 3. Single cell network profiling (SCNP)

Single Cell Network Profiling (SCNP) is a unique proteomic approach that quantifies functional immune signaling capacity, simultaneously across multiple immune cell subsets. One of the major advantages of this technology in the context of tumor immunotherapy is the ability to monitor cellular functional capacity without physical cell isolation. This enables the detection and monitoring of immune signaling and communication within the complex and interlocked immune system. The data generated are highly dimensional, including functional information across many signaling pathways at one time, with resolution down to rare immune cell subsets. This enables the generation of predictive and prognostic information in heterogeneous disease states. Clinical validation of the technology has been established in non-M3 AML, with classifiers for the prediction of response to frontline standard induction therapy in the elderly and pediatric populations [28, 29].

#### 4. Immunohistochemistry

##### PD-L1 level measurement

There is increasing evidence to support the hypothesis that a pre-existent adaptive anti-tumor immune response in the TME correlates with clinical benefit to checkpoint blockade with anti-CTLA-4 or anti-PD-1/PD-L1 inhibitors [30, 31]. Recently, three IHC assays to measure PD-L1 expression have been approved by the U.S. Food and Drug Administration (FDA). One is a companion diagnostic assay to identify advanced NSCLC patients that may be treated with pembrolizumab [32]. The second assay was approved as a complementary diagnostic to inform on risk-benefit for patients with non-squamous NSCLC and melanoma patients treated with nivolumab [33]. The third and most recently approved assay is also a complementary diagnostic that was approved for patients with metastatic urothelial cancer considering treatment with the anti-PD-L1 therapy atezolizumab [34].

Although PD-L1 appears to enrich for response to anti-PD-1/L1 therapy in some disease settings, it has low Negative Predictive Value (NPV), which is of concern in life-threatening diseases such as the end-stage cancer setting, and low Positive Predictive Value (PPV). Adding to the complexity of applying PD-L1 IHC assay in clinical practice is that there are numerous separate diagnostic assays in development, and each might be tied to a different therapeutic agent. Existing tests for PD-L1 detection that have not been FDA approved will require analytical and clinical validation and it is unclear whether the assays will be interchangeable. Consequently, testing the same sample with different PD-L1 assays may yield different results even when used in accordance with the manufacturer’s instructions. The discrepancy in PD-L1 staining using different assays including negative results may be due in part to cellular, spatial, and temporal heterogeneity in PD-L1 expression, which is a dynamic marker of response to T cell activation and it is up-regulated on tumor cells by IFNγ. In addition, differences in antibody usage, various algorithms for scoring as well as cut-off values contribute to the challenge of data interpretation in the clinical setting for this marker.

##### T cell infiltrate

There are indications that an “inflamed” signature in tumors (i.e., the presence of T cell infiltrates) may be associated with improved clinical outcome in response to checkpoint inhibitors as compared with a “noninflamed” phenotype observed in tumors lacking a T cell infiltrate. In addition, significant correlation between the presence of tumor infiltrating lymphocytes (TILs) and the PD-L1 expression in the TME has been described [30].

Pre-treatment samples from melanoma patients who benefited from anti-PD-1 treatment showed a significantly higher density of CD8+ cells at both the invasive margin and the tumor center compared with the group of patients who experienced progression under the same treatment. However, the best predictive parameter for the probability of clinical response to PD-1 blocking therapy was high density of CD8+ T cells at the invasive tumor margin. The next best predictors were CD8+ cells in the tumor center, tumor and invasive margin PD-1 expression, and tumor and invasive margin PD-L1 expression [6]. Classification of tumors into four groups on the basis of their PD-L1 status and presence or absence of TILs has the potential to identify pathways that should be targeted to elicit the best response for each tumor type [35]. Furthermore, clinical responses to checkpoint blockade therapy were found to be associated with T helper type 1 (Th1) gene expression and elevated expression of IFNγ as well as IFNγ-inducible genes [36–38]. Suppressive Tregs and MDSCs may also have a role in negatively affecting the activity of anti-PD-L1-blockade in various tumors [39–41].

The pattern of expression of PD-L1 and tumor inflammation can also differ in tumor subtypes. For example, PD-1/PD-L1 receptors are differentially expressed in molecular subtypes of breast cancer (triple negative breast cancer (TNBC) vs. non-TNBC and colon cancer (CRC) (microsatellite-high (MSI-H) vs. microsatellite stable (MSS) cases). These subsets of immunogenic tumors (e.g., MSI-H CRC) attract TILs, which produce IFNγ that up-regulates PD-L1 on tumor cells and demonstrate characteristic of an inflamed phenotype, such as prominent tumor lymphocytic infiltrate and macrophages located at the invasive front of the tumor. In contrast, most non-inflamed tumors at baseline show a lack of PD-L1 by either tumor cells or tumor infiltrating immune cells. Thus, the presence of T cells and PD-1/PD-L1 can provide an indication for potential benefit of immunotherapy in aggressive subtypes of breast and colon cancers for which no targeted therapy is currently available [42, 43].

It has been previously shown that quantifying the densities of two lymphocyte populations—cytotoxic CD8+ T cells and memory T cells expressing CD45RO+ antigen, CD3+ and CD8+ T cells, or CD3+ and memory CD45RO+ T cells (CD3/CD45RO, CD3/CD8 or CD8/CD45RO)—both in the tumor core and in the invasive margin of tumors, termed “Immunoscore,” could predict survival of early-stage colorectal cancer patients [44, 45]. The prognostic value of the “Immunoscore” is currently undergoing clinical validation as an international effort (NCT01688232). Considering the importance of T cell infiltrate for cancer prognosis, the immune profiling may potentially serve as a predictive biomarker for certain type of immune manipulation, if it can be clinically validated.

Overall, these data suggest that pre-existing adaptive immunity as measured at the tumor level by CD8 T cell infiltration and their spatial distribution as well as PD-L1 expression may be required to predict clinical response to anti-PD-L1 inhibitors. In addition, the presence of Tregs, MDSC, or other T cell inhibitory molecules (such as LAG-3, TIM-3, and IDO) needs to also be characterized to provide a complete view of the interaction between cancer and immune system at the level of the individual patient.

#### 5. Genomic landscape

Recent advances in next generation sequencing (NGS) technologies allow for rapid sequencing of large segments of an individual’s DNA including whole exomes (WES) and entire genome (WGS). NGS technologies utilize high-throughput approaches of clonally amplified or single molecule templates, which are then sequenced in a massively parallel fashion. NGS allows for the identification of a large panel of somatic mutations, i.e., mutational load across different types of cancer. Overall, patients who had tumors bearing a high frequency of somatic mutations like melanoma, NSCLC, and MSI-H colorectal cancer were significantly more likely to achieve clinical benefit from checkpoint blockade including CTLA-4 and PD-1 inhibitors [46–50]. The increased mutation load may activate adaptive immunity and attract CD8+ cell infiltrates, which results in the inflamed tumor phenotype. This suggests that genomic analysis to assess total mutational load could be incorporated in the treatment decision making process to determine who will benefit from immune-therapeutic approaches.

Improvements in computer algorithms to predict neoepitopes from exome sequences that are presented with MHC class I and II as potential targets to T cell receptors will allow further evaluation of the clinical relevance of somatic mutations. These neo-epitopes may aid in the identification of biomarkers to predict overall survival in tumors such as primary lung adenocarcinomas in response to immunotherapy [51]. Putative immunogenic 9– and 10–amino acid neoantigens with affinity for HLA class I molecules using patient-specific nonsynonymous mutations based on HLA types were significantly associated with clinical benefit in some studies [46]. However, the correlation between neoantigen load and clinical benefit diminished when increasingly stringent thresholds for affinity of binding were applied and recurrent neoantigens did not reveal any shared features or features exclusive to responders [52]. These data suggest that clinical relevance of the neoantigens might depend on the proper antigen processing and neoepitope affinity as well as HLA expression, which is frequently aberrant in tumors. Better algorithms might be also needed to assess the immunoprotective properties of mutation derived neoepitopes.

Recent clinical trial data also demonstrated the utility of microsatellite instability (MSI) status as a predictive marker for response to PD-1 blockade in CRC patients treated with a checkpoint inhibitor pembrolizumab [53]. Mismatch repair (MMR) deficiency occurs in a small fraction of CRC as well as cancers of the uterus, stomach, biliary tract, pancreas, ovary, prostate, and small intestine. Tumors with genetic defects in the MMR pathway are known to harbor hundreds to thousands of somatic mutations, especially in regions of repetitive DNA known as microsatellites, which result from deficient MMR machinery. Moreover, MMR–deficient tumors display prominent immune infiltration and Th1-T cells associated cytokine-rich environment as well as immune checkpoint receptors including PD-1 (and its ligand PD-L1), CTLA-4 and LAG-3, a finding consistent with a pre-existent immune response [42, 53–57].

WES of tumor samples followed by extensive bioinformatic analysis to identify immunogenic epitopes is not yet practical for routine diagnostic use. MSI testing, in contrast, is routinely performed in most diagnostic laboratories through the evaluation of selected microsatellite sequences or through an IHC based approach. Therefore, MSI testing has the potential to be an immediately useful approach to predict clinical benefit to PD-1/PD-L1 pathway inhibitors in patients with MMR deficient tumors.

#### 6. Immunosequencing

Immunosequencing is a multiplex PCR-based method that amplifies rearranged TCR complementarity determining region (CDR) 3 sequences for a given TCR locus and exploits the capacity of high-throughput sequencing (HTS) technology to enumerate and quantify hundreds of thousands of TCR CDR3 chains simultaneously. Multiple V, D, and J gene segments exist in the germline genome. Initial receptor diversity is generated by recombination of V, D, and J segments, and additional non-templated diversity is introduced at the junctions by insertion of random nucleotides (N). The immunosequencing assay uses a multiplex PCR with forward primers in each V segment and reverse primers in each J segment. The TCR repertoire from circulating peripheral blood mononuclear cells has been profiled prior to and following administration of an anti-CTLA-4 blocking antibody [58]. In response to the administration of the anti-CTLA-4 monoclonal antibody, there was a marked increase in both the “richness” (number of unique TCRβ sequences) of circulating T cells and the diversity of the T cell population. Interestingly, this increase appeared to be generalized, with no particular clone or subgroup of clones demonstrating a significantly greater increase than others. This observation suggests that clones that have been sequestered or “kept at bay” are somehow released by this therapeutic intervention. Of note, the degree of systemic toxicity associated with this form of therapy also correlated with increases in the richness and diversity metrics, suggesting that some of the clones being kept at bay are those that are capable of conferring more generalized inflammatory or autoimmune responsiveness. Biopsies of skin lesions from patients with metastatic melanoma were obtained and subjected to TCRβ immunosequencing analysis before treatment with anti-PD1 blocking monoclonal antibody [6, 59]. Patients whose tumors had the highest number of T cells and the more clonal T cell repertoire were most likely to respond to this therapy. Conversely, all of those patients whose total T cell number and clonality measure fell below the median for each of these parameters had progressive disease. Moreover, biopsies obtained more than 3 weeks following the initiation of the anti-PD-1 therapy showed that patients whose tumors showed significant expansion of pre-existing T cell clones in response to the therapy were most likely to have demonstrated a clinical response.

#### 7. Multiplexed-gene expression profiling

While the focus of the approaches discussed earlier has been on tumor or immune cells, other technologies assessing predictive biomarkers in the immune-oncology space are focusing on the interaction of tumor cells with the TME including immune cells. Gene expression analysis of RNA levels incorporates a large amount of data that can have prognostic and predictive relevance and can be used to characterize both tumor and immune cells.

The nCounter Dx Analysis system (NanoString Laboratories, Inc.) uses gene-specific probe pairs that hybridize directly with the mRNA in solution eliminating any enzymatic reactions, and does not require RNA amplification that might introduce bias in the results. The nCounter Dx Analysis System assay simultaneously measures the expression levels of up to 800 target genes and a specific panel of immune response genes is also available. The instrument, reagents and software have received 510(k) clearance from the FDA for use with the Prosigna Breast Cancer Prognostic Gene Signature Assay [60].

Considering that there are clinically validated, multi-gene expression prognostic tests currently used in the clinical setting (such as OncoTypeDX, Prosigna, and Mammaprint, the latter two cleared by FDA through the 510(k) process), the probability of gene expression signatures to be developed as markers predicting response to immunotherapy is significant. In this regard, recent data showed that measuring immune-related biomarkers, including T cell specific, antigen presentation–related, and IFNγ signaling–related genes, may allow for improved selection of patients likely to respond to anti–PD-1 therapy with pembrolizumab consistent with the hypothesis that clinical responses to PD-1 blockade occur in patients with a preexisting interferon-mediated adaptive immune response [61, 62].

## Pre-analytical and analytical validation

Although assays for immune-oncology are subject to the same analytical validation requirements as other bio-analytic assays, there are some basic differences that may impact the analytical validation process. Table 2 highlights the differences between single analyte bioassays (measuring a single protein or metabolite) vs. assays measuring immune response. Although immune response assays can be singular, most biomarkers will require multiparameter tests that depend on an increased number of controls, complex scoring algorithms, high-throughput performance data analysis, and results output. In addition, in the US, when a predictive marker will be used to direct patient enrollment or for patient stratification in clinical trials, the assay will need to be performed in a Clinical Laboratory Improvement Amendments (CLIA) laboratory. CLIA labs follow Clinical and Laboratory Standards Institute (CLSI) guidelines for determination of standard assay parameters such as precision, accuracy, limit of detection, specificity, and reference range. A typical analytical validation plan involves several steps in which the assay must be optimized for multiple parameters:

**Table 2:** Comparison of bioanalytical assays with immune response-based assays

Characteristics	Bio-analytical Assays	Immune-based Assays
Number of analytes	Single analyte relative to biological functions (small molecules or macromolecules)	Multiple analytes with complex functional relationships between tumor and immune system
Category of measurement	Absolute quantitation	Quasi-quantitative, relative-quantitative or qualitative. Qantification is available for specific assays
Reference material	Available	Not available, limited availability or available - depends on the assay
Linearity of analyte(s)	Known	Unknown or do not demonstrate linearity, often unknown dynamic ranges, or dynamic range can be established-depends on the assay
Limit of detection (LOD)	Available	Not quantifiable or LOD available - depends on the assay
Sample processing	Extraction required for small molecules; Direct measurement in biological matrix without sample pretreatment	Single cells or specific cell types frequently required for blood-based assay; tissue processing required for FFPE samples; tissue processing required for DNA and RNA
Function assessment	Not necessary - a determinant of the static molecular status	Functional assays often require *ex vivo* response to stimuli
Time for archived clinical samples analysis	Relatively short, <1 yr	Often long >1 yr; Depends on the stability of biomarker/assay

Source: Guidance for Industry: Bioanalytical Method Validation [178]

- Sample-related (pre-analytic parameters)
- Assay-related (analytical parameters)
- Data-related (post-analytical parameters)

### Pre-analytical validation

**Fig. 1:**
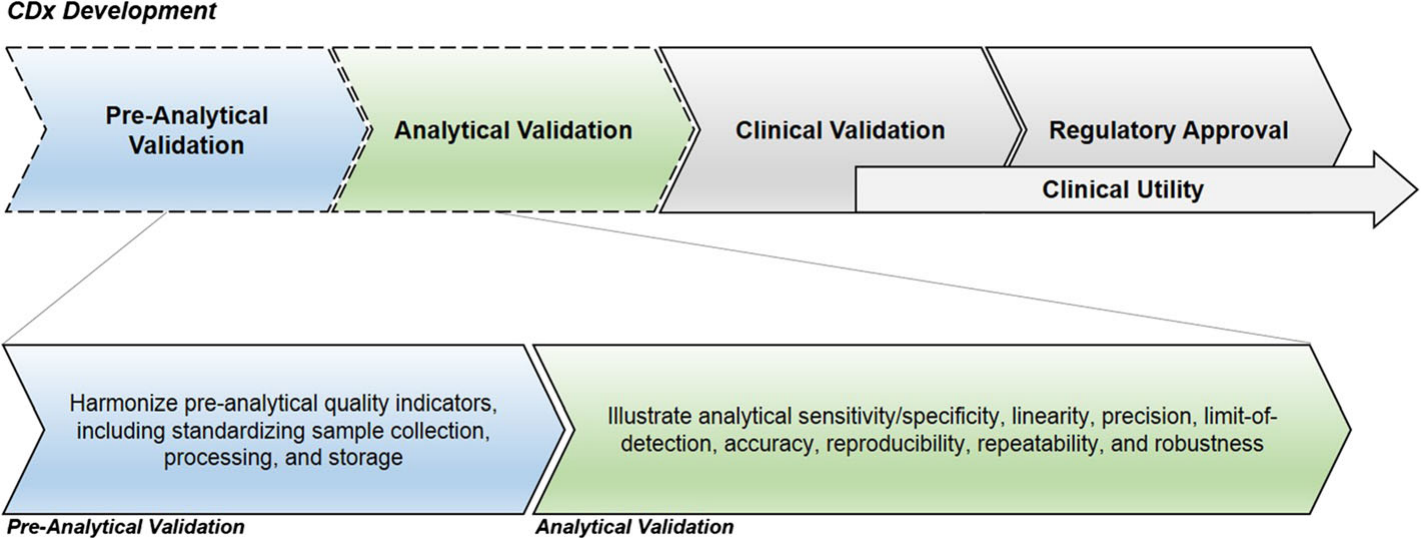
The biomarker development process can be divided into sequential phases, including preanalytical and analytical validation, clinical validation, regulatory approval, and demonstration of clinical utility. This paper focuses on the aspects of the pre-analytical as well as analytical phases of the validation process prior to clinical validation and regulatory approval phases of development. In the pre-analytical phase, pre-analytical quality indicators should be harmonized including sample collection, process, and storage. In the analytical phase, the sensitivity/specificity, linearity, precision, limit-of-detection, accuracy, reproducibility, repeatability, and robustness of the assay must be illustrated

An important step in biomarker validation is the evaluation of pre-analytical factors that may affect assay performance due to specimen-related variability as outlined below (Fig. 1). For immunotherapies, there may be a need to monitor *ex vivo* immune responses in phenotypical or functional assays, which require high-quality samples to ensure reliable analytic output. To ensure that optimal pre-analytic processing regimens are followed, standard operating procedures (SOPs) for controlling specific biomarker development steps are essential. To create the best practice metrics, blood collection and storage media optimization protocols are often developed in conjunction with other pre-analytical parameters. General guidance on pre-analytical quality indicators and their harmonization, including analytical stability and laboratory quality control (QC) have been published [63].

To improve standardization of specimens, the US National Cancer Institute (NCI) has published best practice guidelines for biospecimen collections [64]. In addition, specific guidelines for the analytical requirements of biomarkers have been set up [65, 66].

#### 1. Whole blood and specific immune cell subsets assays

Pre-analytical processing of samples for diagnostic assays including those used for single cell immune response assays, such as ELISpot, flow cytometric analysis, and SCNP, includes patient-related factors such as tissue-ischemia time, pretreatment with drugs, dynamic nature of the analyte, and sample heterogeneity. Analyte stability can be affected by the sample collection process including anticoagulants used for blood draws, freezing/thawing, time between collection and testing, and storage conditions before processing. Guidance documents related to the handling of peripheral blood mononuclear cells (PBMC) has been published previously by the Immunology of Diabetes Society that contains recommendations and references addressing the various pre-analytical steps that need to be considered [67]. Additional guidelines regarding isolation and preservation of PBMC for functional analysis are also available [67–70]. A highly relevant issue for immune-based assays is the avoidance of contamination with granulocytes [71] that are potent suppressors of T cell function in in vitro assays [72, 73]. Processing of fresh whole blood or PBMCs is not always practical in large clinical trials. Thus, cryopreservation of PBMCs is an alternative for the purpose of batching samples over time and for banking samples for future use. However, it can decrease cell viability and function and decrease yield. Therefore, it requires standardization between sites and infrastructure commitment to decrease the variability.

##### Anticoagulants

The optimal anticoagulants chosen to preserve blood samples are highly dependent on the type of target analyte (e.g., nucleic acid or protein), the specific blood cell type of interest (e.g., T cells, B cells, or NK cells), and the specific assay platform. As an example, a study addressing this issue for a gene expression profiling assay resulted in recommendations for Na_2_EDTA over formaldehyde as an RNA stability additive [74] whereas others have found that to preserve cell surface antigen integrity for flow cytometry, sodium heparin was optimal [67]. Special collection tubes, chip-based devices, or media additives for preservation of particular cell subsets are increasingly being deployed to achieve better compatibility with multicenter based late stage clinical trials especially for “liquid biopsy” (circulating tumor cells [75], cell-free DNA [76], and exosomes [77]). These specialized tubes can be prohibitively costly when used in an exploratory banking setting. Thus, in trials testing undefined and exploratory biomarkers, blood cells, serum, and/or plasma may be banked under generalized conditions that may or may not be optimal for a particular analyte and platform.

##### Blood cell components

Immunotherapies targeting specific components of the immune system, e.g., innate, adaptive, memory, naïve cells, and Tregs, can affect both target cells as well as other cells across the immune system. Most of the therapies currently in development engage CD8+ cytotoxic cells, and assessment of cell-mediated cytotoxicity is an important measure to predict immunotherapy response. These therapies might, however, require the development and validation of assays to interrogate other cell subsets for which assays have not been routinely generated, including immune cell subsets such as B cells [78], monocytes/macrophages [79], MDSCs [80], natural killer (NK) cells [81], T helper cells, and other T cell subtypes (Tregs, naïve, and memory T cells) [82].

Different cell subsets require specific pre-analytical protocols, to preserve their cell type-specific functional qualities. To ensure delivery of meaningful results, concurrent assessment of integrity of multiple cell subsets during pre-analytical validation for an optimal combination of parameters (storage, collection, and processing) is highly recommended.

Flow cytometry allows for characterization of many subsets of cells, including rare subsets in a complex mixture such as blood. Flow cytometry can be used to assess not only expression of cell-surface proteins, but also that of intracellular phosphoproteins, cytokines, transcription factors, and functional readouts. The accurate measurements of variation in the human immune system requires precise and standardized assays to distinguish true biological changes from technical artifacts [83]. Because flow cytometry remains highly variable with regard to sample handling, reagents, instruments set up, and data analysis the Human Immunology Project has been proposed for global standardization of flow-cytometry immunophenotyping. In addition, a repository of immunological data for data mining for biomarkers will be part of the project [83].

The ELISpot platform enables analysis of T, B, NK cells as well as of monocytes at the single cell level, though is mainly restricted to the functional aspect of cell analysis. For this platform, PBMC or TILs need to be isolated within a strict time frame to avoid granulocyte contamination and related suppression of functionality [84, 85]. Excellent guidance is provided in the latest CLSI document for the performance of single cell immune assays [69]. Apoptotic cell contamination should be kept to a minimum [86]. Overnight resting of previously frozen samples prior to the assay has been shown to remove apoptotic cells and restore functionality [87, 88].

Multiparametric technology platforms, such as SCNP, enable simultaneous analysis of the functional capacity of multiple and rare immune cell subsets without the need for cell subset isolation or novel sample processing procedures. Samples are drawn into standard sodium-heparin coated tubes, and where necessary, PBMCs are prepared using standard Ficoll separation and cryopreservation procedures for viable sample preparation and storage [89]. Cell-subset identification is performed by *in silico* “isolation” of subsets that are identified by fluorochrome-conjugated antibodies recognizing phenotypic markers.

##### Plasma and serum

Circulating free proteins, chemokine, and cytokine levels can be measured using either plasma or serum samples. Circulating free DNA (cfDNA) in plasma is gaining significance as a monitoring tool for tumor progression and therapy response.

Because major differences exist in the protein profile of plasma and serum, it is important that once chosen as the primary sample type either serum or plasma is consistently used during the entire course of the validation of a blood biomarker test, unless these fluids have been shown to be interchangeable [90]. Common variables to pay attention include: i) the nonlinear dilution pattern of majority of soluble cytokines, ii) preferential distribution behavior of different analyte levels in plasma, and iii) non-specific background that can affect signal reproducibility *via* inhibitory or stimulatory mechanisms. When no one matrix covers every target of interest, thorough validation is highly recommended to define the best matrices to obtain optimal performance, especially under multiplexed setups [91]. For example, IL-6 was found to be significantly less represented in serum than in plasma, while the level of CXCL8 was found higher in serum than in plasma [92]. For individual circulating proteins, chemokine and cytokine, quantitative immunoassays, such as singleplex enzyme-linked immunosorbent assay (ELISA), are frequently used. Multiplex platforms like Luminex or Meso Scale (MSD) technologies are commonly used for quantitation of groups of analytes.

For assay development using biofluids, including cfDNA or miRNA, background effects on the assay readout such as hemolysis should be assessed. The preference is for plasma because the clotting reaction for serum preparation not only alters the proteomic composition of the sample, but also contains DNA from leukocytes and thus is less suitable for tumor specific cfDNA analysis. It is feasible to use samples taken for routine hematology measurements, but lithium heparin tubes should be avoided as lithium is a PCR inhibitor [93, 94]. Consensus SOPs for the collection, processing, handling, and storage of serum and plasma samples for biomarker discovery and validation are available [95].

#### 2. Tissue-based assays

##### Immunohistochemistry (IHC)

Tissue based biomarkers can be measured on freshly frozen (FF) tumor samples or formalin fixed paraffin embedded (FFPE) tissue. FFPE tissue blocks are often available as archival materials as part of bio-banked samples for conventional IHC, which is the most widely used platform for biomarker assessment in diagnostic surgical pathology and for retrospective research. However, damage to the protein and nucleic acid frequently occurs through the fixation, embedding, and prolonged storage of FFPE samples.

IHC is a multi-step process that requires standardized conditions for tissue collection, fixation and processing, preparation of the IHC slide, and interpretation of the staining results. IHC based assays remain important tests as companion diagnostics (CDx) to assess antigen expression on diagnostic or surgical specimens for selecting patients and predicting patient-response to specific targeted therapies (e.g., HER2 expression for Herceptin), and more recently PD-L1 measurement as a CDx for pembrolizumab treatment of NSCLC patients. Published guidelines for measuring established biomarkers such as estrogen receptor, progesterone receptor, and HER2 are available [96, 97]. Of particular importance is the consideration of tissue collection and shipping of paraffin slides, which is a major challenge for multi-institution studies where central processing and banking is performed [98]. General guidelines, including analyte stability and laboratory quality control, for performing analysis of tissue-based molecular biomarkers have been published [99].

Time is a critical factor throughout the biospecimen collection and processing period, especially for proteins that are highly labile. Minimizing the pre-analytic variability for IHC-based analysis needs to address tissue removal from the patient. It is generally accepted that 2 h of ischemia does not significantly alter the protein, DNA or RNA conformation, or preservation of microscopic features. To preserve antigenicity of PD-L1 in IHC assays, it is recommended to store slide-mounted tissue sections in the dark at 2-8 °C. In addition, staining within 6 months of sectioning is recommended for reliable interpretation of PD-L1 expression due to the instability of the antigen [32].

Time to fixation and the fixation period are also critical factors affecting the quality of both RNA and protein, especially phosphoproteins that are notoriously unstable depending on the time of fixation, duration of fixation, and the type of fixative [100]. Published guidelines for optimal protein staining include fixation in 10 % neutral buffered formalin (NBF) for 24 h, dehydration in several changes of xylene and ethanol for 1.5-15 h, and embedding in paraffin for 0.5–4.5 h [101]. For PD-L1 detection, fixation time for 12–72 h in 10 % NBF is recommended, as fixation times of ≤3 h may result in variable PD-L1 detection [32]. The specific conditions, however, may vary from protein to protein due to the biochemical nature of the protein.

Embedding can have a great impact on pre-analytical and analytical variability especially when the presence of tumor immune-infiltrate is required to be integrated in the context of specific location in the tissue specimen, e.g., invasive tumor margin. Association of TILs (e.g., CD3, CD8) at the invasive margin in melanoma has been shown to correlate with response to PD-1 pathway inhibitors [35, 36]. T cell-infiltrate location (invasive margin and/or tumor center) has been previously identified as an important consideration in the “Immunoscore” algorithm for prognosis in CRC and a variety of other tumors [102]. Standardization and consensus guidelines for TILs assessment in breast cancer to foster their integration into future clinical trials and diagnostic practice has also been published [103].

Antigen retrieval conditions also depend on the nature of the antigen and should be carefully controlled (e.g., the pH of the retrieval solution for PD-L1 must be 6.1 ± 0.2, as a pH below 5.9 may give erroneous results). Specific conditions, however, will vary due to the biochemical nature of the antigen, membrane vs. cytoplasmic or nuclear localization as well as variability of expression of the specific antigen in different histologies. To control pre-analytical requirements of the assay’s performance, running the test on a series of in-house tissues with known IHC performance characteristics representing known positive and negative tissues is recommended (reference samples).

Although IHC for a single marker remains a standard method in pathology laboratories, tumor stratification, in particular in immune-oncology, will likely require quantitative and multiple marker approaches to accurately define the multi-dimensional interactions between cancer and the immune system, which are relevant for clinical decision making. A standardized methodology for evaluating PD-L1 expression and TILs might be required as a prerequisite for integrating these parameters in standard histopathological practice as well as in clinical trials. Quantitative and multiplexed IHC and immunofluorescence-based platforms have been discussed in detail in publications resulting from other Biomarker Task Force activities (Additional file 1) [104].

##### DNA-based assays

Next Generation Sequencing (NGS)-based tests for tumor mutation analysis, similar to other complex molecular diagnostics, should demonstrate adequate analytical and clinical performance [105]. They should follow SOPs that specifically address materials and procedures including patient’s sample type, method of DNA extraction as well as technical metrics for DNA quantification and quality, which can negatively impact sensitivity and reproducibility of the assay [106].

For somatic mutation detection using NGS assays, an important pre-analytical consideration is the collection and storage of quality controlled samples. Various standardized preservation methods have been developed for DNA [107] in various sample types including FFPE, FF tissues, and fine-needle biopsies [108, 109]. Nucleic acids, in particular DNA, are more stable than proteins and are therefore less sensitive to variation in sample processing, although formalin fixation has been shown to reduce DNA and RNA solubility and induce a high frequency of sequence alterations [110]. An important factor is determining the minimal amount of FFPE material required for a NGS clinical assay. Usually a minimum of 80 % tumor content in the extracted material from FFPE tumor samples is required, but samples with as low as 10 % of tumor content have been used in research studies [105, 111].

Tumor enrichment using macro-dissection is helpful to quantitatively assess somatic variant allele frequency and copy number values (CNV). It also increases sensitivity and reproducibility of the data. Whole tumor section should be considered when assessing contribution of the tumor stroma, which could be important for quantitation of components of TME including immune system components such as TILs.

The quantity of DNA needed as input for an assay can vary depending on the analyte and assay platform. FFPE tumor DNA from clinical samples presents a challenge for mutation testing specifically when the DNA input from mutated cells is low, the DNA can be damaged, and C > T artifacts in DNA from the fixation and embedding process frequently occur. Amplification steps can be used before sequencing (i.e., library creation), but this process is associated with an increased risk of errors. Quantification of DNA and RNA can be performed by spectrophotometry, fluorimetry, or by PCR. Yet, absorbance does not reflect integrity of DNA since it does not measure fragmentation or degradation resulting from tissue processing. These limitations can be overcome by utilizing novel qPCR type approaches for input material optimization [112].

Immunosequencing of TCRβ for T cell clonality used a multiplex PCR and is routinely performed on genomic DNA extracted from FFPE samples. The size of the amplicon for TCRβ analysis is generally compatible with the level of degradation of DNA caused by the fixation process. Further refinements of the immunosequencing assay to make it even more robust on DNA extracted from FFPE samples are currently under development [113].

##### Gene expression-based tests

The preparation of intact and pure mRNA is one of the key factors in mRNA gene quantification. Extraction of nucleic acids and particularly RNA is very sensitive to nucleases. Thus, nuclease-free conditions should be implemented to control variability in steps such as sample collection, tissue fixation, and FFPE blocks handling including sectioning. For the extraction of nucleic acids from the FFPE tumor tissue, a method for the simultaneous isolation of high-quality DNA, RNA, and microRNA as well as protein from the same sample has been developed [114, 115].

To measure quality, the RNA Integrity Number (RIN) obtained from RNA electropherogram traces (e.g., Bioanalyzer traces) has been used traditionally as measures of FFPE RNA. However, RIN values from degraded FFPE fragments samples are not a sensitive measure of RNA quality and are not reliable predictors for successful library preparation. Illumina developed the DV200 metric to access FFPE RNA quality by accurately measuring the percentage of RNA >200 nucleotides. DV200 > 30 % of RNA samples ensures that degraded RNA fragments meet the requirements for efficient target capture and is a reliable predictor of library preparation [116].

Gene expression analysis using RNAseq, microarrays, or qPCR platforms on RNA prepared from FFPE tissues has been notoriously challenging due to poor quality RNA and the chemical modification of the nucleic acids. Furthermore, assessment of RNA degradation indicates that the degree of RNA fragmentation and the sensitivity to fragmentation depend on the specific transcript. Therefore, selecting a proper internal control gene from listed housekeeping genes for normalization is very critical for successful gene expression analysis using RNAseq analysis. However, other platforms, such as the Nanostring nCounter System, which have been optimized for RNA prepared from FFPE samples do not suffer from the same limitations. Specifically, NanoString probe code-set design and detection method appear to be able to accommodate the fragmented nature of FFPE tissue RNA better than most of the other currently available technologies.

Recent clearance by FDA under 510(k) regulation of the NanoString’s Prosigna (PAM50) gene signature panel showed that when using macro-dissected FFPE tissue slides as the starting sample, the reproducibility was quite high. The analytic validation of a gene expression prognostic signature has been recently published [60]. The analytic studies described in the publication resulted in the optimal tissue and optimal RNA specifications required for acceptance of clinical samples in the marketed assay (i.e., tumor surface area in H&E stained slides >4 mm^2^/slide, tumor cellularity required (>10 %), and need for non-tumor tissue macro-dissection). These data suggest that gene expression profiling upon application of suitable controls and standard procedure can achieve a fit-for-purpose assay for successful clinical application [117].

#### 3. Reagent qualification and stability

One of the crucial steps in the analytic validation of any assay is the qualification of the specific reagents, unique to each test. Chemical compounds can decompose under freeze and thaw cycles, and both short and long term storage conditions can affect cell processing and DNA/RNA extraction. The stability of the stock solutions, of the analyte and the internal standard should be evaluated at assay specific conditions. Conditions used in reagent stability testing should reflect situations likely to be encountered during actual sample handling, storage, and analysis.

As part of the qualification process of assay reagents, stability testing of critical reagents, such as primary antibodies, enzymes, and recombinant cytokines, should be performed to define stability windows and sample expiration dates. Clear directions in prequalification criteria for large-batch stored materials are highly recommended (e.g., a viability cut-off to qualify control donor PBMC used as an in-study quality control). For functional cell-based assays, such as ELISpot and SCNP that require cell-preconditioning, specific validated SOPs ensuring reproducibility are necessary [88, 118].

For example, in order to qualify reagents for SCPN, each antibody-fluorochrome conjugate is titrated independently against 3 qualified control samples to select the optimal titer in the relevant buffer conditions following reagent qualification SOPs. Cocktails comprising all components are then generated following SOPs and before incorporation in the assay are qualified for performance using SOP qualified control samples (cell lines and/or banked control PBMC from healthy donors). Modulators (e.g., cytokines, drugs, anti-TCR, or anti-BCR) are formulated and qualified for assay incorporation using standard samples as for the assay cocktails, testing for both positive and negative signaling (e.g., anti-TCR stimulation should induce signaling in the T cells but not B cells within the well).

### Analytical validation

Analytical validation involves confirming that the assay used for the biomarker measurement has established: i) Accuracy, ii) Precision, iii) Analytical sensitivity, iv) Analytical specificity, v) Reportable range of test results for the test system, vi) Reference intervals (normal values) with controls and calibrators, vii) Harmonized analytical performance if the assay is to be performed in multiple laboratories, and viii) Establishment of appropriate quality control measures. The requirements for analytical validation as well as their definitions are summarized in full in Table 3.

**Table 3:** Analytical validation requirements for biomarker assays

Requirement	Definition
Analytical sensitivity	The ability of the assay to distinguish the analyte of interest from structurally similar substance
Analytical specificity	The degree of interference by compounds that may resemble but differ from the analyte to be quantified
Linearity	The ability of an assay to give concentrations that are directly proportional to the levels of the analyte following sample dilution
Precision	The agreement between replicate measurements
Limit of detection	The lowest concentration of analyte significantly different from zero, also called the analytical sensitivity
Accuracy	Agreement between the best estimate of a quantity and its true value
Repeatability	Describes measurements made under the same conditions
Reproducibility	Describes measurements done under different conditions
Robustness	Precision of an assay following changes in assay conditions, e.g., variation in ambient temperature, storage condition of reagents

Source: Jennings et al., 2009 [179]

#### 1. Precision

Analytic repeatability and reproducibility is a requirement for the implementation of all diagnostic tests and is particularly critical for predictive assays given the implications of misclassifications of patients for treatment. Use of positive and negative controls and standardized SOPs are required to assure reproducibility. Guidelines for the number of replicates needed to validate the performance of molecular diagnostic assays, as well as such considerations as the linearity of assay response, dynamic range, limits of detection, analyte stability within the intended matrix, and intra- and inter-laboratory coefficient of variability have been provided [19, 119].

Precision refers to closeness of agreement between a series of measurements and evaluates random error that may be identified as within-run, between-run within-day, between-day, or within-laboratory. Precision is quantitatively expressed in terms of the standard deviation (SD), variance, or coefficient of variation (CV) of a series of measurements. Precision is often a function of the analyte concentration, with small concentrations resulting in poorer precision (i.e., larger SD, variance, and CV) than high concentrations. Precision should be assessed at the medical decision points of relevance to the intended clinical application of the tumor biomarker. Precision is determined by reproducibility and repeatability of the assay which allow quantitative determination of the closeness of agreement among measurements. The reproducibility is generally measured by the % CV, which is defined as the standard deviation divided by the mean of the assay result expressed as a percent [65].

The FDA and European Medicines Agency (EMA) acceptance criteria for biological assays typically define the required between-run and within-run precision as CV of 10 or 15 % for quality control samples and 20 % for lower limit of quantification (LLOQ) samples [120, 121]. However, the new CLSI guidelines for single cell-based functional assays suggests larger CV acceptance (up to 30 %) and requires more repetitions (6 to 10 replicates) in assay validation to reflect the high degree of heterogeneity of the majority of live cell-based immune assays (including intracellular cytokine staining, HLA-peptide multimer assay, ELISpot, and cell proliferation assays) [69]. It is important to note that the ultimate CV acceptance can only be evaluated in the clinical context in which the test is used (e.g., for a patient stratification assay variability around the test cutoff together with the distribution in the target patient population of the test results will need to be considered).

Depending on the particular category, an assay can require a distinct type of analytic validation. Definite quantitative assays make use of calibrators and a regression model to calculate absolute quantitative values for unknown samples. The reference standard must be well defined and should be a representative of the biomarker. This type of assay can be accurate and precise. In relative-quantitative assays, reference calibrators can be used; however, because standards are not fully representative of the biomarker, assay precision can be validated, while the accuracy of the assay can only be estimated.

Precision for single cell immune assays, e.g., ELISpot (including intra- and inter-assay variability as well as reproducibility) is a particularly critical validation parameter. Inherent variability of these assays should be adequately addressed as they are frequently used in the clinic to longitudinally monitor changes in immune parameters in response to an immune intervention (such a vaccine administration). Precision data are essential to render results of measurements at different time points that are comparable in a meaningful way i.e., an increase in the magnitude of measured responses after vaccination/treatment has to significantly differ from the determined variability. Precision testing includes replicate measurements of the same conditions in one experiment (repeatability) and repetition of the assay with the same samples on different days by all assay operators involved in a study (intra-assay precision) and in all participating laboratories (reproducibility), if applicable.

A rather challenging task with these assays is to determine accuracy, i.e., the closeness of agreement of the measured value and the true value. This is particularly true for ELISpot as well as for other single cell functional assays, due to the lack of a gold standard/test that is able to provide an exact measurement of antigen-specific cells in a given sample. Obtaining data on how accurate a laboratory performance is in relation to a specific assay can be achieved *via* participation in large proficiency panels that provide relative accuracy for a laboratory in comparison to other laboratories testing the same sample(s) in the same assay. An international ELISpot Proficiency Panel for IFNɣ is conducted on a yearly basis and is open for participation to any laboratory independent of affiliation or research background [122].

Efforts to harmonize classic single-cell immune monitoring assays have included the identification of critical assay steps, and guidelines for harmonized assay conduct have been made available (ELISpot [123–125], multimer staining [126, 127], intracellular cytokine staining [128–130] and Immunoscore [131, 132]). These efforts have been shown to dramatically reduce the variability among laboratories and provide a basis for the comparison of immune assay results obtained at different sites, or even across trials [133].

For SCNP, captured data include quantification of cell subset frequencies and specific intracellular read outs for each of the cell subsets in both the basal (unmodulated) and modulated state. In addition, various aspects of modulated signaling in each cell subset and/or signaling inhibition by in vitro drug exposure are captured by metrics that are computed by comparing data for cells subject to different conditions. In this manner, the degree of evoked signal, for example, is established by comparing data obtained in the modulated well for a specific donor sample with the data obtained from the same sample in the adjacent unmodulated well. The “Fold” metric is applied to measure magnitude of the responsiveness of a signal in a specific cell population relative to the unmodulated reference. The proportion of a cell population that is responsive to modulation is measured by the Uu (rank based metric based on Mann–Whitney U statistic) metric. Similarly, inhibited signaling is captured using both magnitude and population-based metrics [118]*.*

Reproducibility of semi-quantitative assays such as IHC is a unique problem in that it is difficult to measure variation between assay results. For IHC assays, results are usually expressed as low, medium, or high or on a scale of 1 to 3. For such assays, reproducibility is generally measured in terms of the kappa (ĸ) statistic and percent agreement among different observers [134]. Although there is no generally accepted value of ĸ that indicates the level of agreement, it has been suggested that ĸ <0.4 represents poor, 0.4–0.6 moderate, 0.6–0.8 significant, and 0.8 very good agreement: total agreement is indicated by a value of 1.0 [135].

A semi-quantitative assays do not use calibration standards but has a continuous response that is expressed in terms of a characteristic of the test sample. Precision can be validated but not accuracy. The ideal level of agreement or concordance in such assays is unclear, although a level of agreement of 85 % is considered to be acceptable. Inter-observer reproducibility might represent a major challenge to the reliable assessment of the IHC results in addition to tissue-processing.

#### 2. Multiparametric assays

Validation and maintaining reproducibility of multiparametric assays is much more challenging considering the number of analytic variables associated with high content assays (such as NanoString, flow cytometry, SCNP, mutational load, and TCR sequencing). The capacity of high-throughput platforms, such as nCounter Dx Analysis System (NanoString) or flow cytometry based analysis SCNP enable multi-dimensional analysis of the immune system. Instead of detecting a single or limited number of molecular targets, assays are able to detect tens to hundreds of distinct molecular features simultaneously [136].

SCNP enables the simultaneous analysis of the functional capacity of multiple immune cell subsets in the same well. Controls for assay performance, reagents, and multiplexing are therefore required to validate reproducibility and precision [118]. Multiplexed reagent “cocktails” are generated comprising 8 or more fluorochrome-conjugated antibodies that recognize both cell surface and intracellular phenotyping molecules (e.g., CD3, CD4, CD56, and FoxP3) and intracellular readouts of activity (e.g., p-Akt, and p-ERK) following sample modulation with selected stimuli. The use of pre-formatted lyophilized-reagent plates (Lyoplates, BD Biosciences) can help to decrease staining variability compared with using individual liquid reagents in multiple studies in immunophenotyping [137] as well as functional assays [138].

To control for multiplexing, each assay should be run with a well-characterized control for assay performance included in the top row of every plate (healthy control donor PBMCs or cell line). In addition, rainbow control particles included in the final column of each plate should be included to control for cytometer performance and enable normalization within and across plates. The control samples (typically healthy donor PBMCs) are typically from leukapheresed whole blood in which multiple vials of the same donor preparation are available and are qualified for use following a standard signaling panel defined by SOPs. Control donor bridging across assays is also performed where appropriate. When cell lines are used, batch preparations are made to cover multiple assay runs and are qualified following SOPs.

For NGS, assay performance characteristics include: accuracy (degree of agreement between the nucleic acid sequences derived from the assay and reference sequence); precision (the degree to which repeated sequence analyses give the same results); repeatability (within-run precision); reproducibility (between-run precision); and sensitivity (the likelihood that the assay will detect the targeted sequence variations, if present). Sensitivity also includes the probability that the assay will not detect a sequence variation when none is present. Two different NGS platforms using different chemistries for amplification based systems coupled to massively parallel sequencing are commonly used for NGS applications (Illumina TruSeq and Ion Torrent AmpliSeq). Each platform has specific parameters relevant to the laboratory and test requirements including instrument size, instrument cost, run time, read length, and cost per sample [116, 139, 140].

For WES and WGS, the focus of validation is on developing metrics that define a high-quality exome/genome, such as the average coverage across the exome/genome and the percentage of bases that meet a set minimum coverage threshold. The minimum acceptable level of the concordance of single nucleotide polymorphisms (SNPs) identified as compared with the reference should be established). Minimum coverage threshold necessary to determine variants relevant for the diagnostics need to be also established experimentally as low coverage increases the risk of missing low-level variants. Even after the macro-dissection step, patient tumor samples are still contaminated with normal cells derived from surrounding tissue or from reactive infiltrate, which may skew the representation of mutant alleles. The American College of Medical Genetics (ACMG) has developed clinical laboratory standards for NGS [106], which specifically address the unique challenges of WES/WGS [141].

The TCR immunosequencing assay is a Laboratory Developed Test (LDT) that has been CLIA and CAP certified. Data presented at the time of these certifications supported the following assay parameters: analytic accuracy, sensitivity, lower level of detection (LOD), lower limit of quantification (LLOQ), specificity (including interfering factors), linear reportable range, and precision.

Two methods have been analytically validated to determine MSI phenotype in colon cancer, yet neither is FDA approved/cleared. PCR analysis with a panel of mononucleotide markers (BAT-25, BAT-26, MONO-27, NRhwe21, and NR-24) and IHC based analysis of the MMR proteins (MLH1, MSH2, MSH6, and PMS2) have been proposed. Both tests show high reproducibility; however, IHC-based test, unlike PCR, has disadvantages such as dependence on antibody panels and challenges of analytical performance evaluation of the IHC based assay. CAP provides a detailed summary on several clinically important issues, such as the number and types of markers used, methods used to perform the assay, and definition of MSI-H and MSI-L phenotypes. This information is valuable to clinical laboratories that are currently offering this test as well as to those that are planning to launch this test for predicting response to anti-PD-1 inhibitors [142, 143].

#### 3. Reference materials for immune assays

For efficient assay development, particular care must be given to establish the conditions that allow validation of the assay to meet required sensitivity and specificity by usage of well-defined standards. Inclusion of appropriate control materials to ensure that assays are working accurately and reproducibly is a key to the success of any assay. Each experiment must include controls that reflect both the analytical and post-analytical processing to assess artefactual findings leading to misinterpretation of experimental results. Ideally, consistent reference materials should be used across all stages of analytical validation. Table 4 provides a list of recommended standard materials as reliable controls for specific immune assays. There are two different types of reference materials depending on the purpose of application: i) validation references and ii) quality control references.

**Table 4:** Recommended standard materials for immune assays

Technology/Assay	Recommended Reference Materials
Blood-based assays	Cell lines or control PBMC donor samples prepared and cryopreserved following SOP (reference samples)
Immunohistochemistry assays	Control cell lines or tissue specimens (e.g., human tonsil for PD-L1 staining) or cell lines processed and embedded in paraffin (not to be used for interpreting patient data)
RNA/DNA based assays (NGS, TCR/BCR sequencing, gene expression profiling)	Synthetic mimic libraries, TCR/BCR DNA synthetic templates, synthetic vectors, company or WHO and NIST references

Reference materials are used in assay validation to estimate intra- and inter-run accuracy/precision and stability. Quality control reference materials are used during in-study sample analysis to accept or reject assay runs. For both types of reference materials, low (undetectable, <LOD) and high (maximum working concentration) reference levels can be established as negative and positive controls, respectively. The same biological sample can serve multiple purposes (e.g., as validation reference and quality control reference). However, a validation reference, by its nature, is used to show assay parallelism with patient samples, behaving with similar performance measurements (i.e., specificity, precision, and sensitivity), while the quality control references are used to test acceptance criteria.

Because of the lack of well-characterized and well-regulated “reference standard materials” (typically authorized by US Pharmacopeial Convention (USP) and National Institute of Standards & Technology (NIST) or other international agencies such as National Institute for Biological Standards and Controls (NIBSC), World Health Organization (WHO), etc.) for quantitative measures of immune analytes, reference materials often in the forms of biological samples are used to assess relative accuracy of an assay performance (cell lines and tissue specimens). To better reflect the complexity of immune cell-based assays, synthetic reference materials or “home-brew” references are created by preparing mixtures of known analyte(s) (e.g., recombinant proteins) at known concentrations.

Unlike quantitative assays in which the result is a continuous number expressed using an approved or certified reference standard, semi-quantitative assays, such as immune response assays, rarely have reference standards and are expressed in relation to a baseline characteristic of a sample. These assays generally lack calibrators but may have standards for the different categorical values that are usually not certified by a regulatory body.

For blood-based assays, the reference samples may include cell lines or control PBMC donor samples that are prepared and cryopreserved following SOPs to ensure standardized preparation. These controls are qualified for use following SOPs that define both the test and the required output data parameters for inclusion in the assay. For example, in SCNP a defined range of signaling across pre-specified nodes is used to qualify a sample for use as a control. The use of PBMC from leukapheresed whole blood enables the generation of large batches of control donor PBMC that can cross multiple assay runs. For T cell assays, specific TCR-engineered T cells can be obtained and used as performance control [144]. “Bridging” samples are used to enable the transfer of one control donor to another over time and multiple assay runs in instances where one donor sample would be exhausted.

For IHC, cores containing positive and negative protein expressing or genetically modified  cell lines that are extensively characterized using molecular assays, IHC, Western blot and fluorescent in situ hybridization (FISH) or well characterized tissue specimens are recommended to be included on the same slide. For example, human tonsil tissue is recommended for PD-L1 IHC as strong positive staining should be detected in portions of the crypt epithelium and weak to moderate staining of the follicular macrophages in the germinal centers. Negative staining should be observed in endothelium, fibroblasts, and surface epithelium [32]. Cultured cell lines could represent an alternative source of material for quality control that are homogenous, uniform in quality, and can be processed and embedded in paraffin. Culture cell lines can be used as a control for the validity of the staining, but should not be used for interpretation of patients’ data [145]. Efforts using validation of RNA levels for accurate PD-L1 detection is also ongoing [146].

Although relative quantitative assays constitute the great majority of immune response assays so far, RNA or DNA-based methods, such as NGS, TCR sequencing or gene expression profiling methods that may become predictive for response to immunotherapy, if validated, are highly quantitative due to availability of synthetic reference materials. Generally, major sequencing reagent providers have a set of standards that serve to control instrument performance in addition to standards for technical performance of the assay in order to conserve reads for clinical samples in a run.

The NIST recommended HapMap NA12878 control is used for standardization of platform performance when the data are compared with the well-curated, publically available data from different consortia, e.g., Genome in a Bottle (GIAB) Consortium for NA12878, which has extensively quality-controlled reference standard materials for analytical validation of NGS platforms, including DNA standard reference materials with high accuracy for whole genome sequences [147].

In the case of FFPE tissue-based tests for somatic mutations, control DNA samples available from companies, such as Horizon Dx or Acrometrix (Thermo Fisher, Inc.), provide controls with a clear readout of variant calls at defined positions that greatly aid in the development of somatic mutation assays. Use of controls that match anticipated specimens (such as FFPE controls) in addition to high quality, non-formalin fixed cellular HapMap control materials like NA12878 is particularly useful for establishing background error for formalin-fixation caused deamination based errors, e.g., high background of C/T variant calls and other fixation based artifacts as well as calculation of index calling efficiency with pipelines being utilized [105].

The immunosequencing assay makes use of independently chemically synthesized templates for every possible V and J combination for any locus for which the assay is developed [148]. These templates provide a known set and frequency of rearranged sequences that allow for control of PCR-bias. They serve as internal controls for every reaction that is run. They can be distributed by a third party regulatory concern for use in laboratory proficiency testing.

Examples of synthetic reference materials also include synthetic vectors serving as reference to control amplification bias for DNA, and cDNA-based NGS, “alien” sequences (sequences of nucleotides which do not exist in humans) as negative controls for the nCounter platform [148].

### Post-analytical criteria

The post-analytical phase of biomarker evaluations involves data interpretation of the assay results. Dichotomous variables are relatively straightforward to incorporate into calculations of data sensitivity and specificity. However, most variables in measurement of immune response are continuous, resulting in variability with respect to analytical performance criteria and clinical relevance of the assay, e.g., cutoff points for clinical decision making. Essentially, a cutoff for classifying a sample as positive or negative needs to be determined empirically by correlating results with clinical outcomes in a clinical trial exploring efficacy of a drug as discussed in Volume II.

Flow cytometry-based data interpretation considers many different aspects such as pre-defined gating and clustering strategies, choice of appropriate data transformation for data visualization, inclusion and exclusion criteria, and so on, as shown by numerous published harmonization efforts [129, 149–151]. The minimal reporting guidelines for biological and biomedical investigations (MIBBI) project include a series of reporting frameworks (http://mibbi.sourceforge.net/foundry.shtml) to guide scientific publishing and data reporting to specific web sites where independent analysis is possible. There are several “minimal information” sub-projects under assay or platform-specific focus groups. Flow cytometry (MIFlowCyt) [152] and T cell assays (MIATA) [153], NK cell assays (MIANKA), and FISH assay (MISFISHIE) [154] are those most relevant to immune status monitoring. These initiatives provide useful suggestions for scientific data reporting and may help researchers to determine the degree of laboratory details captured.

Immunohistochemical methods are notoriously nonlinear, and scoring systems are generally vulnerable to heterogeneity in intensity extent and topography of staining. Because of a lack of universal methods, scoring systems for IHC are usually based on characteristics of overall staining intensity using a scale of 0 to 3+ and subcellular localization [119]. The main pitfalls of PD-L1 as a predictive biomarker may be related to both the variability in expression due to tumor heterogeneity as well as IHC assay variability due to different antibody clones, staining platforms, scoring systems, and clinical sampling points. These factors increase the uncertainty for using PD-L1 expression as a patient selection biomarker. Together, these challenges may contribute to the low NPV and PPV of PD-L1 as a predictive marker of clinical benefit to anti-PD-1/PD-L1 blockade.

**Fig. 2:**
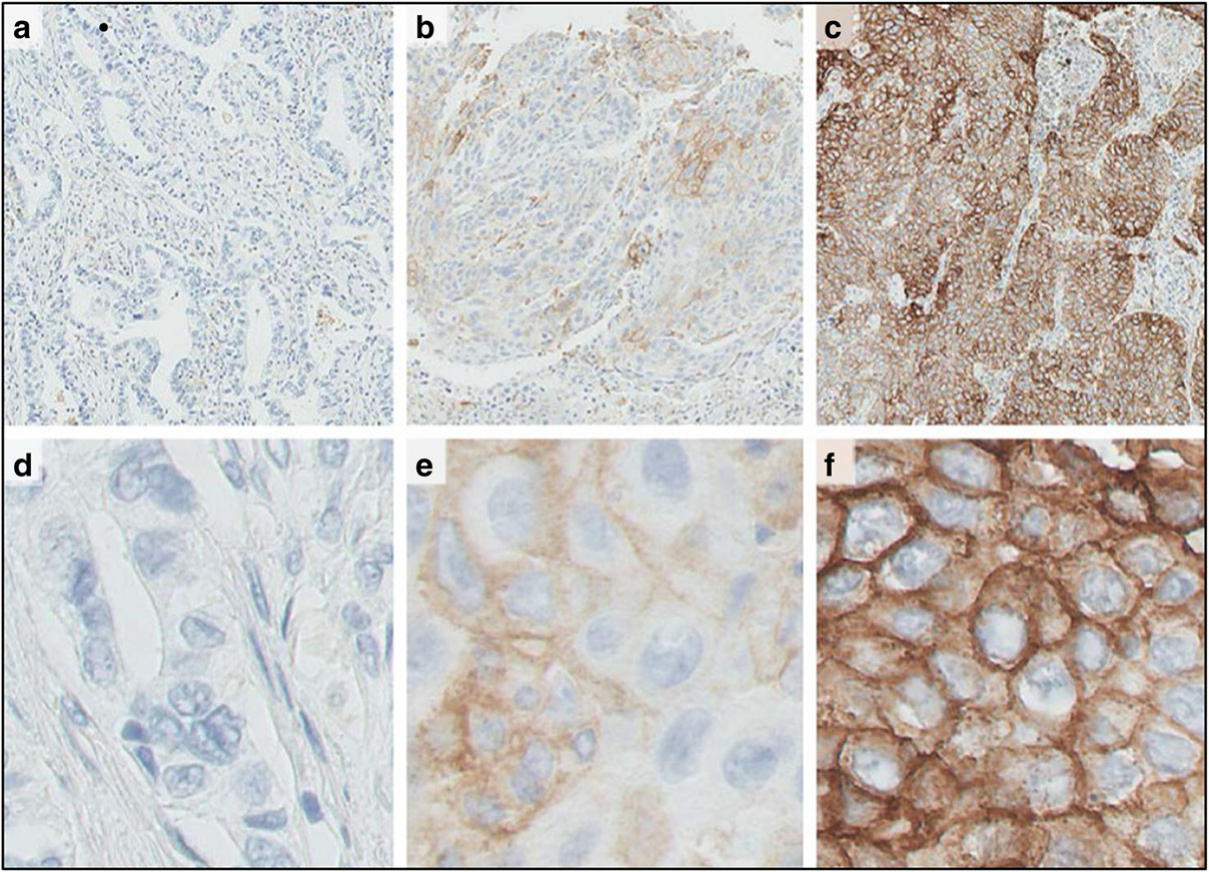
PD-L1 Expression in Non–Small-Cell Lung Cancers. Results were reported as the percentage of neoplastic cells showing membranous staining of programmed cell death ligand 1 (PD-L1) (proportion score). Shown are tumor samples obtained from patients with a proportion score of less than 1 % (Panel **a**), a score of 1 to 49 % (Panel **b**), and a score of at least 50 % (Panel **c**) (all at low magnification). Tumor samples with the corresponding proportion scores are shown at a higher magnification in Panels **d** through **f**. PD-L1 staining is shown by the presence of the brown chromogen. The *blue* color is the hematoxylin counterstain. From The New England Journal of Medicine, 2015, 372, 2018-2028 Edward B. Garon et al., Pembrolizumab for the Treatment of Non–Small-Cell Lung Cancer. Copyright © 2015 Massachusetts Medical Society. Reprinted with permission from Massachusetts Medical Society

There are numerous drugs in development targeting the PD-1/PD-L1 pathway; the practice has been to independently develop anti-PD-L1 IHC CDx for individual agents. The different PD-L1 IHC diagnostic kits and assays vary in different percentages of positive cells, scoring systems, and cutoff values (from 1 to 50 %), cells scored (tumor cells and/or infiltrating immune cells), and in the subcellular localization of staining (membrane vs. cytoplasmic). If each therapeutic was approved in conjunction with a specific CDx, this may present a challenge for testing and decision making in the clinic. Examples of tumor samples with different percentage of tumor cells staining for PD-L1 are shown in Fig. 2. PD-L1 immunostaining with a percentage of tumor cell staining of 50 % or higher was associated with significantly longer progression-free survival and overall survival than a lower than 50 % percent of stained cells in a KEYNOTE 001 trial with pembrolizumab in NSCLC. If each therapeutic was approved in conjunction with a specific CDx, this may present a challenge for testing and decision making in the clinic.

Thus, the FDA, the American Association for Cancer Research (AACR),and American Society of Clinical Oncology (ASCO) convened a workshop titled “Complexities in Personalized Medicine: Harmonizing Companion Diagnostics Across a Class of Targeted Therapies” to address comparability across multiple PD-L1 tests. A highlight of the workshop was the unveiling of a “blueprint” proposal developed by four pharmaceutical companies (Bristol-Myers Squibb, Merck & Co. Inc., AstraZeneca PLC, and Genentech, Inc.) and two diagnostic companies (Agilent Technologies, Inc./Dako Corp and Roche/Ventana Medical Systems, Inc.) to analytically cross-compare the four different diagnostics [155]. The scope of this study was to establish technical comparability and to define the key performance parameters of each assay. Preliminary results of this effort were presented at the 2016 AARC annual meeting. Analyses from the Blueprint Project confirm that there is high concordance for the two approved PD-L1 diagnostics in NSCLC [156].

Because IHC is the cornerstone of hospital pathology, significant efforts to measure T cell immune infiltrates as potential predictive markers for clinical decision-making in immunotherapy have been focused in particular on multiplex quantitative IHC approaches. Image-based readouts for IHC using automated methods remove the subjectivity of the traditional system and provide more continuous and reproducible scoring of protein expression in tissue samples. The assessment of TILs by digital image analysis has the potential, for example, to determine the number of TILs per mm^2^ stromal tissue as an exact measurement contrary to the approximate semi-quantitative evaluation currently used. Automated quantitative analysis (AQUA) provides an automated IHC-based analysis and scoring system for assessing the target protein’s signal intensity normalized over the tumor areas and subcellular compartment of biological significance [157]. AQUA has been noted as a promising new strategy for the measurement of hormone receptors testing in breast cancer tissue [158, 159].

Recently developed mass cytometry techniques with the ability to allow multiplexed and directly quantitative imaging of tissue samples helps to overcome many of the current IHC limitations. In these approaches, primary antibodies labeled with rare lanthanide metals with a unique mass that is easily assessed by time-of-flight mass spectrometry. Imaging software is used to re-construct the 2-D stained tissue image from the detected heavy metal ions. CyTOF (Cytometry by Time-Of-Flight) utilizes a laser to destroy the tissue/antibodies and free heavy metal ions. A two dimensional image is created that looks very similar to a routine IHC but with quantitative multiplexed information [160]. Multiplexed ion beam imaging (MIBI) uses a scanning ion beam to liberate the metal ions, which improves the resolution but requires more specialized setup (vacuum, multiple detector MS) [161]. These methods will likely allow for quantitative approaches and development of models to integrate vast amounts of immune response-related information and apply it into clinically applicable settings.

Given the huge amount of sequence data produced by NGS platforms, the development of accurate and efficient data handling and analysis pipelines is essential. NGS data analysis can be divided into four primary operations: (i) base calling, (ii) read alignment, (iii) variant calling, and (iv) variant annotation. A very large number of algorithms are available for each discrete step in data analysis. The accuracy of identifying variants greatly depends on the depth of sequence coverage and variant call quality scores vary between algorithms because of the weighting of quality scores for surrounding bases as well as positional context with respect to primer position and stretches of repetitive bases. Therefore, the final list of quality filtered base calls can be quite different when the same raw data is subjected to analysis with different data analysis software. Another common discrepancy between variant callers involves reporting only non-synonymous and deleterious mutations while other analysis provide a complete list of mutations without filtering for synonymous, coding vs. non-coding, and deleterious vs. tolerated mutations [105].

For NGS bioinformatics pipelines, a very large number of algorithms are available for each step in data analysis to assess the quality of raw NGS data available for whole exome data analysis, including data preprocessing, alignment, post-alignment processing, variant calling, annotation, and prioritization tools. Starting from available exome sequencing data, mutations can then be assessed for their immunogenic potential in the context of each patient’s MHC haplotype using epitope prediction algorithms. These algorithms provide an estimate of the total number of mutation-associated neoantigens in each tumor. Although the number of predicted mutation-associated neoantigens is usually small, it might be proportionate to the number of actual mutation-associated neoantigens, and tumors with a high number of actual mutation-associated neoantigens are more likely to stimulate the immune system to react against the tumor [51, 162, 163].

In the NanoString platform, the nSolver™ Analysis Software is a validated data analysis program for automatic QC, normalization, and data analysis. It performs automated background subtraction corrections; implements customized quality control on samples/lanes, runs the predictive algorithm, and provides customized sample/patient reports.

As high-throughput methods became widely available there is a need for computational methodologies for interpretation of the complex data for biological and clinical implications. Algorithms to develop multimodal signatures integrating various types of molecular tumor data (i.e., genomics, protein expression, and functional analyses) with TME factors that reflect the complex biomarker information require the development of multifactorial classifiers/algorithms. A list of commonly used bioinformatics tools for different high-throughput technologies have been provided and discussed in other publications from the SITC Immune Biomarkers Task Force activities [104].

Any software used to automate any part of the assay for clinical application must ultimately be validated for its intended use prior to clinical application, as required by 21 CFR §820.70(i) [164]. In addition, computer systems used to create, modify, and maintain electronic records and to calculate multiplexed assay results (e.g., outputs of algorithmic models) are also subject to the same validation requirements.

Such computer systems must be validated to ensure accuracy, reliability, consistent intended performance, and the ability to discern invalid or altered records. Testing of device software functionality in a simulated use environment and user site testing are typically included as components of an overall design validation program for a software automated device. In large measure, software validation is a matter of developing a “level of confidence” that all requirements and user expectations for the software automated functions and features of the device are met.

## Conclusions

The biological complexity of the tumor and immune system interaction contributes to multiple challenges associated with technical development of clinically applicable assays when evaluating different variables as markers of clinical benefit to immunotherapy. Recent developments in research and technologies have facilitated better understanding of this interaction and will provide means for development of such assays. However, each of the potential biomarkers and the associated assay demands high-quality validation so it can reach clinical application. To date, various promising candidate assays and platforms to predict response to immunotherapy are available, as discussed in this publication and other reports of the SITC Immune Biomarkers Task Force activity (Additional file 1). However, so far, only the PD-L1 IHC assays to inform anti-PD-1/PD-L1 treatment have been validated for clinical utility. Considering the increased relevance and emphasis on biomarker development in cancer immunotherapy, there is an enormous need to facilitate and improve the steps to demonstrate clinical value of molecular diagnostics in this space. Although many guidelines for assay validation are available, this review differs from previously published reports, as it covers the key steps in the entire process including: i) analytical validation (Volume I), ii) clinical validation, iii) the strategies for demonstration of clinical utility and iv) the regulatory approval process for clinically applicable diagnostics (Volume II) in the context of assays for immunotherapy response. Applying approaches and recommendations as outlined in this review should enable more efficient assay development to identify biomarkers, which are crucial to guide personalized therapy and for advancing immunotherapy options for cancer patients. Therefore, the implementation of the following practices/steps are recommended:

1. Ensure a fit-for-purpose approach for assay development, including biomarker selection and validation.
2. Specific quality-control and quality assurance practices for appropriate procurement for blood-based and the tissue-based assays for each specific biomarker should be considered.
3. Ensure that optimal pre-analytic processing regimens and standard operating procedures (SOPs) for controlling specific biomarker are followed.
4. Procedures with rigorous quality assurance, reproducibility, and control procedures built in should be considered for analytical validation step.
5. The interpretation of assay results must be complemented by proper reference standards, including reagents and assay controls (positive and negative controls, if appropriate).
6. Biostatistics and computerized approaches for data quantification and interpretation as well as algorithm development for multiplex signatures based on phenotypic, functional, and genomic data should be considered.
7. Bioinformatics approaches for the integration of complex, multicomponent, high-throughput types of molecular data from tumor and immune factor analysis should be considered.
8. To evaluate the robustness of semi-quantitative methods and to enable the analytical and clinical validation of biomarkers, reference standards and/or coordinated efforts across centralized laboratories (proficiency panels) are recommended.

**Recommended guidelines**

- General Guidance for Fit-for-purpose Biomarker Validation [19]
- Best Practices for Biospecimen Resources, NCI, NIH [64]
- List of Cleared or Approved Companion Diagnostic Devices, FDA [165]
- Regulations of General Biological Products Standards, FDA [121]
- Guidance for Gene Expression Profiling Platforms, FDA [117]
- Standards for Next Generation Sequencing [168, 169]
- Principles of Analytical Validation for Immunohistochemical Assays [167]
- Guidelines for Validation of Cell Based Fluorescence Assays [170]

CLSI documents

- Guidelines for Evaluation of Qualitative Test Performance [181]
- Guidelines for Evaluation of Precision Performance of Clinical Chemistry Devices [180]
- Guidelines for Verification of Precision and Estimation of Bias [166]
- Guidelines for Quality Assurance for Immunohistochemistry [145]
- Guidelines for Performance of Single Cell Immune Response Assays [69]
- Guidelines for Enumeration of Immunologically Defined Cell Populations by Flow Cytometry [182]

## Additional file

Additional file 1: Publications by the SITC Immune Biomarker Task Force. (DOCX 14.1 kb)

## Abbreviations

AACR: American association for cancer research
AEC: Absolute eosinophil counts
ASCO: American society of clinical oncology
BCR: B cell receptor
CAP: College of American pathologists
CAR: Chimeric antigen receptor
CDR: Complementarity determining region
CDx: Companion diagnostic
cfDNA: Circulating free DNA
CLIA: Clinical laboratory improvement amendments
CLSI: Clinical and laboratory standard institute
CNV: Copy number values
CTLA-4: Cytotoxic lymphocyte-associated antigen 4
CV: Coefficient of variation
ELISA: Enzyme-linked immunosorbent assay
ELISpot: Enzyme-linked immunospot
EMA: European medicines agency
FDA: Food and drug administration
FF: Freshly-frozen
FFPE: Formalin-fixed, paraffin-embedded
FISH: Fluorescent in situ hybridization
GIAB: Genome in a bottle
GM-CSF: Granulocyte-macrophage colony-stimulating factor
HTS: High-throughput sequencing
ICCS: International clinical cytometry society
IFN: Interferon
IHC: Immunohistochemistry
ISLH: International society for laboratory hematology
mAb: Monoclonal antibody
MDSC: Myeloid derived suppressor cells
MIBI: Multiplexed ion beam imaging
MMR: Mismatch repair
MSI: Microsatellite instability
NBF: Neutral buffered formalin
NCI: National cancer institute
NGS: Next generation sequencing
NIBSC: National institute for biological standards and controls
NIST: National institute of standards and technology
NK: Natural killer cell
NPV: Negative predictive value
NSCLC: Non-small cell lung cancer
OS: Overall survival
PBMC: Peripheral blood mononuclear cells
PD-1: Programmed cell death protein 1
PD-L1: Programmed cell death ligand 1
PPV: Positive predictive value
QC: Quality control
RCC: Renal cell carcinoma
RIN: RNA integrity number
SCNP: Single cell network profiling
SITC: Society for immunotherapy of cancer
SOP: Standard operating procedure
TCR: T cell receptor
Th1: T-helper type 1
TILs: Tissue infiltrating lymphocytes
TMA: Tissue microarrays
TME: Tumor microenvironment
TNBC: Triple negative breast cancer
TNF: Tumor necrosis factor
Tregs: Regulatory T cells
USP: US Pharmacopeial convention
WES: Whole exome sequencing
WG: Working group
WGS: Whole genome sequencing
WHO: World Health Organization
